# The validity and applicability of CAD-RADS in the management of patients with coronary artery disease

**DOI:** 10.1186/s13244-019-0806-7

**Published:** 2019-12-04

**Authors:** Mohammad Abd Alkhalik Basha, Sameh Abdelaziz Aly, Ahmad Abdel Azim Ismail, Hanan A. Bahaaeldin, Samar Mohamad Shehata

**Affiliations:** 1 Department of Radio-diagnosis, Faculty of Human Medicine, Zagazig University, Zagazig, Egypt; 2 Department of Radio-diagnosis, Benha University, Benha, Egypt

**Keywords:** Computed tomography angiography, Coronary artery disease, Reproducibility of results, Clinical decision-making

## Abstract

**Background:**

The coronary artery disease reporting and data system (CAD-RADS) is designed for a uniform standardization of coronary computed tomography angiography (CCTA) reporting and further management recommendations of coronary artery disease (CAD). This study aimed to assess clinical validity, applicability, and reproducibility of CAD-RADS in the management of patients with CAD.

**Methods and results:**

A single-center prospective study included 287 patients with clinically suspected or operated CAD who underwent CCTA. Four reviewers evaluated the CCTA images independently and assigned a CAD-RADS category to each patient. The invasive coronary angiography (ICA) was used as the reference standard for calculating diagnostic performance of CAD-RADS for categorizing the degree of coronary artery stenosis. The intra-class correlation (ICC) was used to test the inter-reviewer agreement (IRA). Reporting was provided to referring consultants according to the CAD-RADS. Based on ICA results, we have 156 patients with non-significant CAD and 131 patients with significant CAD. On a patient-based analysis, regarding those patients classified as CAD-RADS 4 and CAD-RADS 5 for predicting significant CAD, the CAD-RADS had a sensitivity, specificity, and an accuracy of 100%, 96.8 to 98.7%, and 98.3 to 99.3%, respectively, depending on the reviewer. There was an excellent IRA for CAD-RADS categories (ICC = 0.9862). The best cutoff value for predicting significant CAD was > CAD-RADS 3. Eighty-seven percentage of referring consultants considered CAD-RADS reporting system to be “quite helpful” or “completely helpful” for clinical decision-making in CAD.

**Conclusion:**

CAD-RADS is valuable for improving CCTA structural reports and facilitating decision-making with high diagnostic accuracy and high reproducibility.

## Key points

- CAD-RADS has an excellent performance for categorization of coronary artery disease (CAD).
- There is an excellent inter-reviewer reproducibility for CAD-RADS categories.
- Referring consultants considered CAD-RADS a helpful classification for clinical decision-making in CAD.

## Background

Coronary artery disease (CAD) is the most common cause of death worldwide, and thus, early recognition is essential to avoid its related complications and improve prognosis [1]. Invasive coronary angiography (ICA) is considered the gold standard for the anatomical evaluation of CAD, but an invasive and costly procedure, with periprocedural morbidity and mortality [2]. Recently, coronary computed tomography angiography (CCTA) has emerged as a powerful modality to exclude obstructive coronary artery stenosis in the diagnostic workup of patients with suspected CAD [3]. Using CCTA has increased because of its non-invasive nature, the high negative predictive value in ruling out significant CAD, and recent technological advances [4].

Because of the discrepancy and high variability in CCTA reporting, a structured CAD evaluation template has long been required [5]. The structured reporting system for CCTA was introduced in 2016 by Curye et al. and is called the “coronary artery disease reporting and data system” (CAD-RADS) [6]. It is the first attempt to provide a simple, concise, and accurate classification of CAD. This new classification is expected to improve the communication and facilitate understanding of the CCTA report with the added inputs of further investigations and management recommendations. Although many institutions across the world have been using CAD-RADS over the last 2 years, it is still not widely accepted [7]. Moreover, the clinical impact on patient prognosis has not been extensively studied [8].

Several recent studies [9–11] have examined the diagnostic validity of CCTA in the evaluation of CAD. However, no prior research has evaluated such validity in reference to the CAD-RADS classification system. Consequently, we performed the current prospective study to estimate the diagnostic validity, applicability, and reproducibility of CAD-RADS for predicting patients at high risk for significant CAD, and assess the value of such classification for decision-making in clinical settings.

## Methods

### Study design and population

**Fig. 1:**
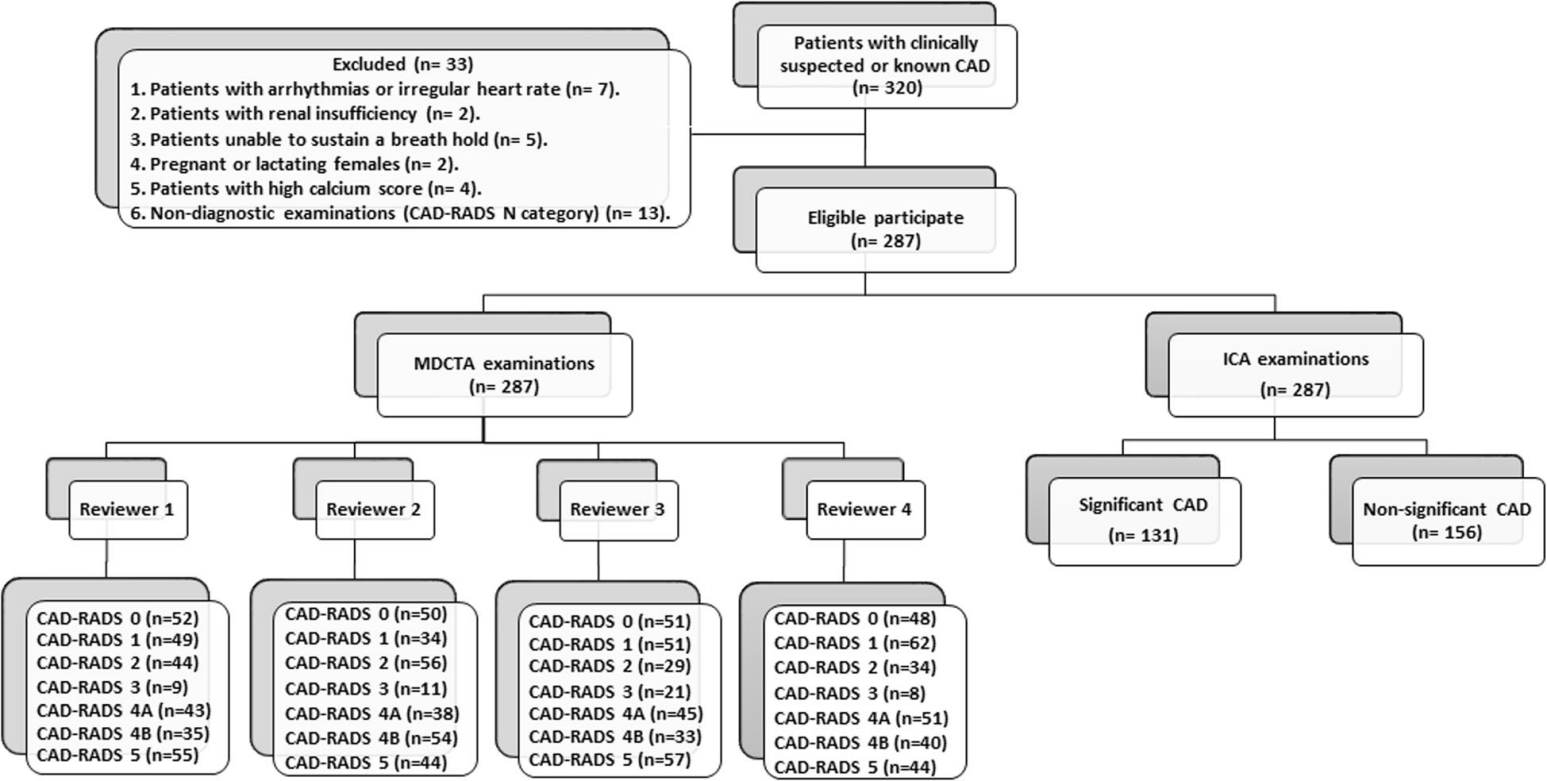
Study flow chart. The study flow chart shows the number of included and excluded patients, the CAD-RADS categories, according to the reviewer, and the invasive coronary angiography (ICA) results

This is a single-center prospective study. Approval was obtained from the institutional review board, and all participants provided written informed consent. We applied the ethical concepts of the Declaration of Helsinki during planning for this study. Between January 2017 and December 2018, 320 consecutive patients with clinically suspected CAD were recruited. Inclusion criteria were *(i)* adult patients’ ≥ 18 years, *(ii)* patients with normal heart rate, and *(iii)* symptomatic patients with clinically suspected CAD or with previous coronary revascularization. Exclusion criteria were *(i)* patients with arrhythmia or irregular heart rate (*n* = 7), *(ii)* patients with renal insufficiency (*n* = 2), *(iii)* patients unable to sustain a breath-hold (*n* = 5), *(iv)* pregnant or lactating females (*n* = 2), *(v)* patients with high calcium score (Agatston score > 1000) (*n* = 4), and *(vi)* non-diagnostic examinations (CAD-RADS *N* category) (*n* = 13). The final cohort of our study included 287 patients (191 males and 96 females; mean age 53.1 ± 10.8 years; range 28–72 years; mean body mass index 31.7 ± 5.9 kg/m^2^). One hundred seventy-eight (62%) of our patients were high risk, 79 (27.5%) were an intermediate risk, and 30 (10.5%) were low risk. A high percentage (33.1%) of our patients were treated with a stent or bypass graft. The patients’ data are summarized in Table 1. The flow chart of our study is illustrated in Fig. 1. Once enrolled, all patients were submitted to contrast-enhanced retrospective ECG-gated CCTA scan and ICA for diagnosis of CAD.

**Table 1:** Patients’ data

Demographics	Values
Age (years), mean ± SD (range)	53.1 ± 10.8 (28–72)
Sex, male/female	191/96
BMI (kg/m^2^), mean ± SD (range)	31.7 ± 5.9
Pretest probability	
High risk	178 (62)
Intermediate risk	79 (27.5)
Low risk	30 (10.5)
Cardiovascular risk factors	
Hypertension	237 (82.6)
Diabetes mellitus	66 (23)
Dyslipidemia	196 (68.3)
Current smoker	71 (24.7)
Family history of premature CAD	151 (52.6)
Clinical presentation	
Typical chest pain	78 (27.2)
Atypical chest pain	61 (21.3)
Non-specific chest pain	148 (51.5)
Patients with stent or bypass graft	
Stent	17 (5.9)
Graft	78 (27.2)
Agatston calcium score, mean ± SD (range)	331.2 ± 465.7 (0–1600)
Ejection fraction by CCTA, mean ± SD (range)	48.8 ± 9.4 (33–67)

Unless otherwise indicated, data are number with the percentage in parenthesis

*BMI* body mass index, *CAD* coronary artery disease, *ICA* invasive coronary angiography, *SD* standard deviation, *CCTA* coronary computed tomography angiography

### Protocol of CCTA

#### Patient preparation

All steps of the examination were explained in detail to each patient. Patients were ordered to fast for 4–6 h before examination without discontinuity of their medications and to prevent caffeine and atropine for 12 h. For avoiding respiratory motion artifacts, respiratory training of breath-holding for 15–20 s with hand placed on the epigastric region must be carefully tested. Unless contraindicated, a beta-blocker (100–200-mg metoprolol) was administrated orally 1 h before the scan to maintain heart rate < 65 beats/min. Ensure a proper connection of the ECG machine to the gantry and leads. After finishing the examination, the patient is kept under observation for 15 min to check the vital signs (pulse and blood pressure). In our institute, we no longer use sublingual isosorbide dinitrate before the CCTA, as we noticed that multiple patients developed tachycardia that worsen the examination, and others suffered from hypotension and severe headache after the examination.

#### Image acquisition

A 128-detectors scanner (Philips Healthcare Ingenuity) was used in all examinations. First, a non-contrast scan was done for calcium scoring; if there were excessive coronary calcifications (Agatston score > 1000), the examination was aborted, and if none, a retrospective ECG-gated angiographic scan was started using a bolus-tracking technique. The scan extended from the carina down to the infra-diaphragmatic level. A bolus of 100 ml (1.5 ml/kg) of warmed nonionic contrast media (Ultravist, 350–370 mg of iodine per ml) was injected through an 18–20-gauge cannula in the antecubital vein at 5–5.5 ml/s flow rate, followed by 50 ml saline flush to reduce the streak artifact at superior vena cava. The injection was performed using a programmed dual-head power injector pump. When the density within the descending aorta reached 180 to 220 HU, image acquisition was begun. The scan was obtained during a single breath-hold using the following parameters: 100–120 kV, 800 mA, 128 × 0.6-mm collimation, and 0.6-mm pitch. The total scanning time was approximately 10–12 s. Ejection fraction was evaluated by CCTA and echocardiogram.

### Image reconstruction and interpretation

All CCTA images were transported to the workstations, and image analysis was conducted on the PACS system (PaxeraUltima—paxeramed) or a dedicated platform Extended Brilliance Workstation (Philips Medical System, Best, The Netherlands). The axial images were reviewed to ensure good image quality and assess cardiac and thoracic anatomy. A 0.6-mm slice thickness was applied for image reconstruction techniques, which included multiplanar reformation (MPR), curved MPR, three-dimensional maximum intensity projection (3D-MIP), and 3D-volume rendering technique (3D-VRT). All CCTA examinations were revised and interpreted by four experienced reviewers with over 5 years of experience in cardiac imaging. All reviewers were blinded to patients’ clinical data and reports of ICA. Before the study started, each reviewer was provided by several clinical sessions of lecture-based on the details of CAD-RADS as well as hands-on instruction session with some practical cases other than the study population.

The following features obtained at CCTA were individually evaluated for each patient: *(i)* coronary calcification (total Agatston calcium score), *(ii)* origin, course, termination, and dominance of the coronary arteries, *(iii)* size of coronary arteries and degree of luminal stenosis (the percentage of stenosis was quantitatively measured by using a specific software), *(iv)* plaque characterization, *(v)* signs of plaque vulnerability (low-attenuation, spotty calcification, positive remodeling, napkin-ring sign) [12, 13], *(vi)* stent/grafts: course/site, patency, and complications (in known CAD patients with previous revascularization), and *(vii)* non-coronary cardiac and extra-cardiac findings. Segmental analysis of the coronary arteries was performed for arteries > 1.5 mm in diameter according to the society of cardiovascular computed tomography (SCCT) model on a per-patient basis [14].

Each reviewer independently assigned a CAD-RADS category for each patient using the CAD-RADS classification system developed by the SCCT (Table 2) [6]. Additionally, each reviewer was also required to assign CAD-RADS modifiers, including the presence of a vulnerable plaque (V), bypass graft (G), and coronary stent (S) for each patient.

**Table 2:** Grading of coronary artery stenosis and management recommendations of symptomatic patients with CAD

CAD-RADS category	Stenosis grade (%)	Management
0	Normal (0)	-No further evaluation of ACS is required. Consider other etiologies.
1	Minimal (1–24)	-Consider evaluation of non-ACS etiology. -Consider referral for out-patient follow-up for preventive management of coronary atherosclerosis and risk factors modification.
2	Mild (25–49)	-Consider evaluation of non-ACS etiology. -Consider referral for out-patient follow-up for preventive management of coronary atherosclerosis and risk factors modification.
3	Moderate (50–69)	-Consider hospital admission with cardiology consultation, functional testing, and/or ICA for evaluation and management. -Recommendation for anti-ischemic and preventive management should be considered as well as risk factor modifications. Other treatments should be considered if there is the presence of hemodynamic significant lesion.
4	Sever (A—70–99%) (B—left main > 50% or 3-vessel obstructive disease)	-Consider hospital admission with cardiology consultation and further evaluation with ICA and revascularization is appropriate. -Recommendation for anti-ischemic and preventive management should be considered as well as risk factor modifications
5	Occluded (100)	-Consider expedited ICA on a timely basis and revascularization if appropriate. -Recommendation for anti-ischemic and preventive management should be considered as well as risk factor modifications.

*ACS* acute coronary syndrome, *CAD* coronary artery disease, *CAD-RADS* coronary artery disease reporting and data system, *ICA* invasive coronary angiography

Before initiating the study, several clinical sessions were offered to referring clinicians (cardiologists, cardiothoracic surgeons, and internal medicine consultants with over 15 years of experience) explaining the meaning and goal of CAD-RADS. Additionally, a management protocol according to CAD-RADS recommendations (Table 2) [15] was provided to referring consultants aiming to determine whether this reporting system could be helpful for deciding patient management and in avoiding confusion for clinicians.

### Reference standard

The diagnoses of CAD were confirmed based on the ICA results. ICA was done as per referring consultants’ request. All ICA examinations were performed within 2 months after CCTA using 6-French high-flow Judkins catheters (Cordis, Miami, FL, USA), and a quantitative validated coronary angiographic system (Philips Azurion3, Philips Healthcare, Netherlands), via a computer-assisted semi-automated edge detection algorithm. Three experienced interventional cardiologists (with over 15 years of experience) performed and analyzed all ICA examinations. All interventional cardiologists were blinded to the CCTA reports. Images were acquired in multiple projections, and the degree of coronary stenosis was evaluated in two orthogonal views to determine the significance of CAD. The final diagnosis of the degree of stenosis was obtained by a consensus. Based on ICA results, our patients were classified categorically as non-significant CAD (< 70% stenosis) and significant CAD (≥ 70% stenosis).

### Statistical analysis

The collected data were computerized and statistically analyzed using MedCalc program (version 11.1; MedCalc, Mariakerke, Belgium). Continuous variables are shown as mean ± standard deviation (SD), while categorical variables are shown as frequency with percentages. We used a four-fold table test to estimate the diagnostic performance of CAD-RADS classification for predicting patients with significant CAD, considering CAD-RADS 0, 1, 2, and 3 as non-significant CAD and CAD-RADS 4 and 5 as significant CAD. The intra-class correlation (ICC) statistic for multiple reviewers was applied to assess overall inter-reviewer reproducibility of CCTA findings and CAD-RADS scoring results. The ICC values were interpreted as follows: 0.01–0.20 = poor agreement; 0.21–0.40 = fair agreement; 0.41–0.60 = moderate agreement; 0.61–0.80 = good agreement; and 0.81–1.0 = excellent agreement. The correlations were calculated using Pearson’s correlation coefficient and presented as *r* and *p* value. The receiver operating characteristic (ROC) curve was applied to calculate the cutoff value and the area under the curve (AUC). To determine how helpful they found the CAD-RADS classification for understanding CCTA reports and making decisions about patient management, referring consultants were requested to complete a simple survey. This survey included a single question: “How helpful do you consider the CAD-RADS classification system is for understanding CCTA reports and providing confidence in your clinical decisions?” and there were five available answers: *(i)* completely helpful; *(ii)* quite helpful; *(iii)* neither helpful nor useless; *(iv)* useless; *(v)* completely useless.

## Results

The current study enrolled 287 patients with suspected CAD. We successfully performed all CCTA and ICA examinations without any side effects (the mean time from CCTA to ICA was 37 ± 12.3 days). Based on ICA results, we have 156 patients with non-significant CAD (CAD-RADS 0, 1, 2, and 3), and 131 patients with significant CAD (CAD-RADS 4 and 5). The prevalence rate for significant CAD was 45.6%.

### Assignment of CAD-RADS categories and modifiers

Categorization of CAD and modifiers based on CAD-RADS classification system is detailed in Table 3. A high percentage (33.1%) of our patients were treated with a stent or bypass graft. The modifier G (bypass graft) was the most common modifier in our patients (27.2%).

**Table 3:** Frequency distributions of CAD-RADS categories and modifiers for 287 patients stratified by reviewers

CAD-RADS categories and modifiers	Reviewer 1	Reviewer 2	Reviewer 3	Reviewer 4
CAD-RADS categories				
0	52 (18.1)	50 (17.4)	51 (17.8)	48 (16.7)
1	49 (17.1)	34 (11.8)	51 (17.8)	62 (21.6)
2	44 (15.3)	56 (19.5)	29 (10.1)	34 (11.9)
3	9 (3.1)	11 (3.9)	21 (7.3)	8 (2.8)
4A	43 (15)	38 (13.3)	45 (15.6)	51 (17.8)
4B	35 (12.2)	54 (18.8)	33 (11.5)	40 (13.9)
5	55 (19.2)	44 (15.3)	57 (19.9)	44 (15.3)
Total	287 (100)	287(100)	287(100)	287(100)
CAD-RADS modifiers				
S (stent)	17 (5.9)	17 (5.9)	17(5.9)	17 (5.9)
G (bypass graft)	78 (27.2)	78 (27.2)	78 (27.2)	78 (27.2)
V (high-risk plaque)	9 (3.1)	13 (4.5)	11 (3.8)	5 (1.7)
Total	104 (36.2)	108 (37.6)	106 (36.9)	100 (34.8)

Data are number with the percentage in parenthesis

*CAD-RADS* coronary artery disease reporting and data system

### Diagnostic performance of CAD-RADS for predicting CAD

On a patient-based analysis, the diagnostic performance of CAD-RADS for predicting significant CAD is summarized in Table 4. Regarding those patients classified as CAD-RADS 4 and CAD-RADS 5 for predicting significant CAD, the CAD-RADS had a sensitivity, specificity, and an accuracy of 100%, 96.8 to 98.7%, and 98.3 to 99.3%, respectively, depending on the reviewer.

**Table 4:** Diagnostic performance of CAD-RADS for patient-based detection of significant CAD

	Reviewer 1		Reviewer 2		Reviewer 3		Reviewer 4	
	%	95% CI	%	95% CI	%	95% CI	%	95% CI
Accuracy	99.3		98.26		98.61		98.26	
Sensitivity	100.00	97.22 to 100.00	100.00	97.22 to 100.00	100.00	97.22 to 100.00	100.00	97.22 to 100.00
Specificity	98.72	95.45 to 99.84	96.79	92.68 to 98.95	97.44	93.57 to 99.30	97.44	93.57 to 99.30
AUC	0.99	0.98 to 1.00	0.98	0.96 to 1.00	0.99	0.97 to 1.00	0.99	0.97 to 1.00
Disease prevalence	45.64	39.78 to 51.60	45.64	39.78 to 51.60	45.64	39.78 to 51.60	45.64	39.78 to 51.60
Positive predictive value	98.50	94.67 to 99.82	96.32	91.63 to 98.80	97.04	92.59 to 99.19	97.04	92.59 to 99.19
Negative predictive value	100.00	97.63 to 100.00	100.00	97.59 to 100.00	100.00	97.60 to 100.00	100.00	97.60 to 100.00

*AUC* Area under curve, *CAD* coronary artery disease, *CAD-RADS* coronary artery disease reporting and data system, *CI* confidence interval

### Inter-reviewer agreement for CCTA findings and CAD-RADS scoring results

Inter-reviewer agreement (IRA) for CAD-RADS scoring results is presented in Table 5. The subanalysis of IRA by categories was moderate to good (ICC = 0.4728–0.7653), with CAD-RADS 3 category as the least concordant (ICC = 0.4728). The overall agreements for CAD-RADS categories and modifiers were excellent (ICC = 0.9862 and 0.8064, respectively).

**Table 5:** Inter-reviewer agreement for CCTA findings and CAD-RADS scoring results

Feature	Inter-reviewer		
	ICC	95% CI	*p*
CAD-RADS categories			
0	0.6977	0.5376 to 0.8124	< 0.001
1	0.6901	0.5421 to 0.7991	< 0.001
2	0.7609	0.6457 to 0.8457	< 0.001
3	0.4728	0.2649 to 0.6864	< 0.001
4A	0.7416	0.6047 to 0.8396	< 0.001
4B	0.7653	0.6325 to 0.8556	< 0.001
5	0.6606	0.4904 to 0.7844	< 0.001
Total	0.9862	0.9834 to 0.9886	< 0.001
CAD-RADS modifiers	0.8064	0.7391 to 0.8598	< 0.001
Vulnerable plaque features	0.5453	0.1962 to 0.8732	< 0.001

*CCTA* coronary computed tomography angiography, *CAD-RADS* coronary artery disease reporting and data system, *ICC* intra-class correlation, *CI* confidence interval

### ROC analyses

**Fig. 2:**
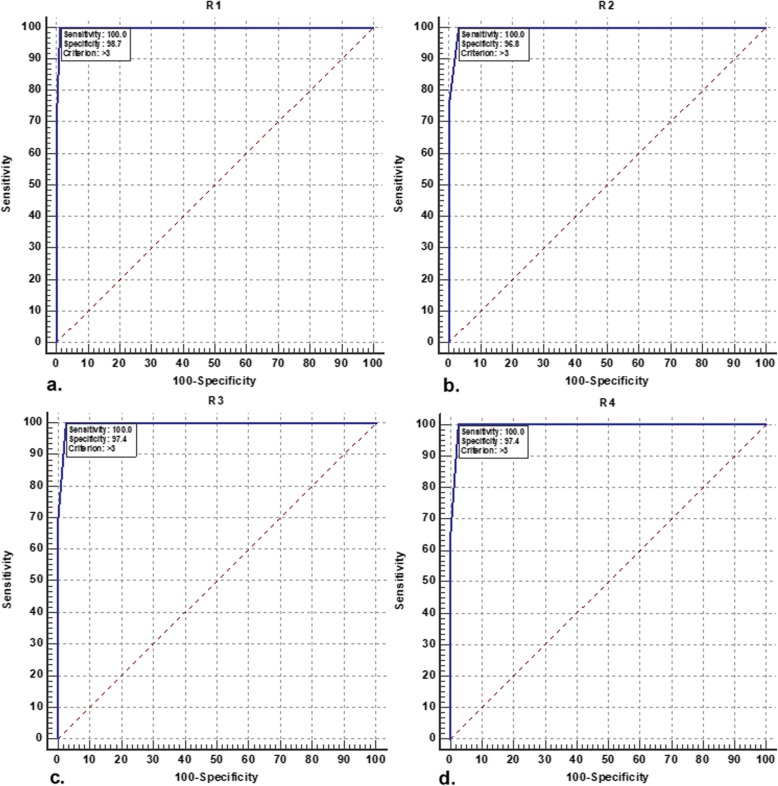
The ROC analyses of multiple reviewers for the diagnostic performance of the CAD-RADS for predicting significant CAD as evidenced by ICA as a reference standard. **a** Reviewer 1. **b** Reviewer 2. **c** Reviewer 3. **d** Reviewer 4. The best cutoff was > CAD-RADS 3

We analyzed the data set of the diagnostic performance of CAD-RADS to determine the cutoff value for predicting significant CAD using the ROC curve depending on the reviewer (Fig. 2). Based on ROC analyses, all reviewers agreed that the optimal cutoff value for predicting significant CAD was < CAD-RADS 3. The use of this cutoff value was associated with AUC ranges from 0.995 to 0.998 (95% CI, 0.979 to 1.000, *p* <  0.0001), sensitivity of 100% (95% CI, 97.2 to 100.0), and specificity ranges from 96.8 to 98.7% (95% CI, 92.7 to 99.8), depending on the reviewer.

### Referring consultant survey results

Twenty-one (87.5%) out of 24 referring consultants considered CAD-RADS reporting system to be “quite helpful” or “completely helpful” for clinical decision-making in CAD. Two found it neither helpful nor useless, and one found it useless.

**Fig. 3:**
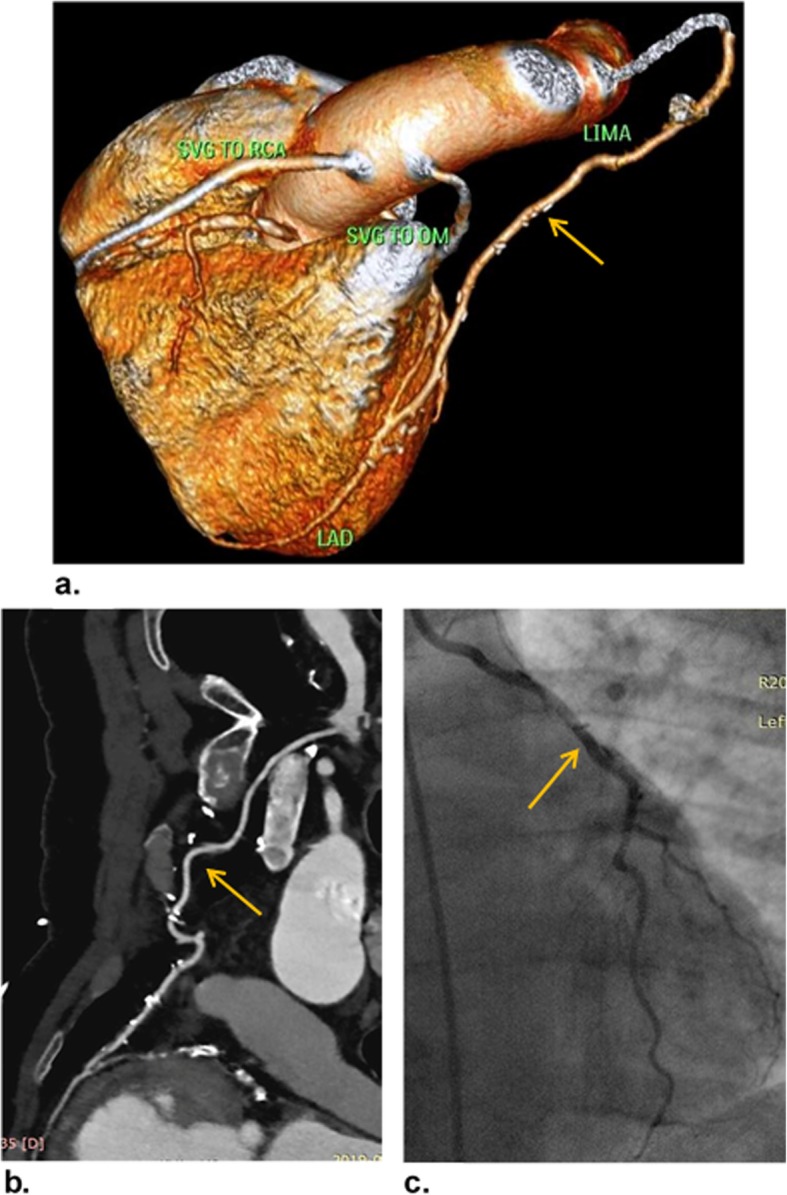
A 54-year-old man underwent CABG 7 years ago, referred for follow-up CCTA. **a** VR image shows patent LIMA-LAD, SVG-OM, and SVG-RCA grafts (orange arrow). **b** Curved MPR image confirms the patency of LIMA-LAD graft along its whole length with patent proximal and distal anastomotic sides as well as patent native LAD distal to the graft (orange arrow). The patient was categorized as CAD-RADS 0/G. **c** ICA image confirms the patency of LIMA-LAD graft (orange arrow)

**Fig. 4:**
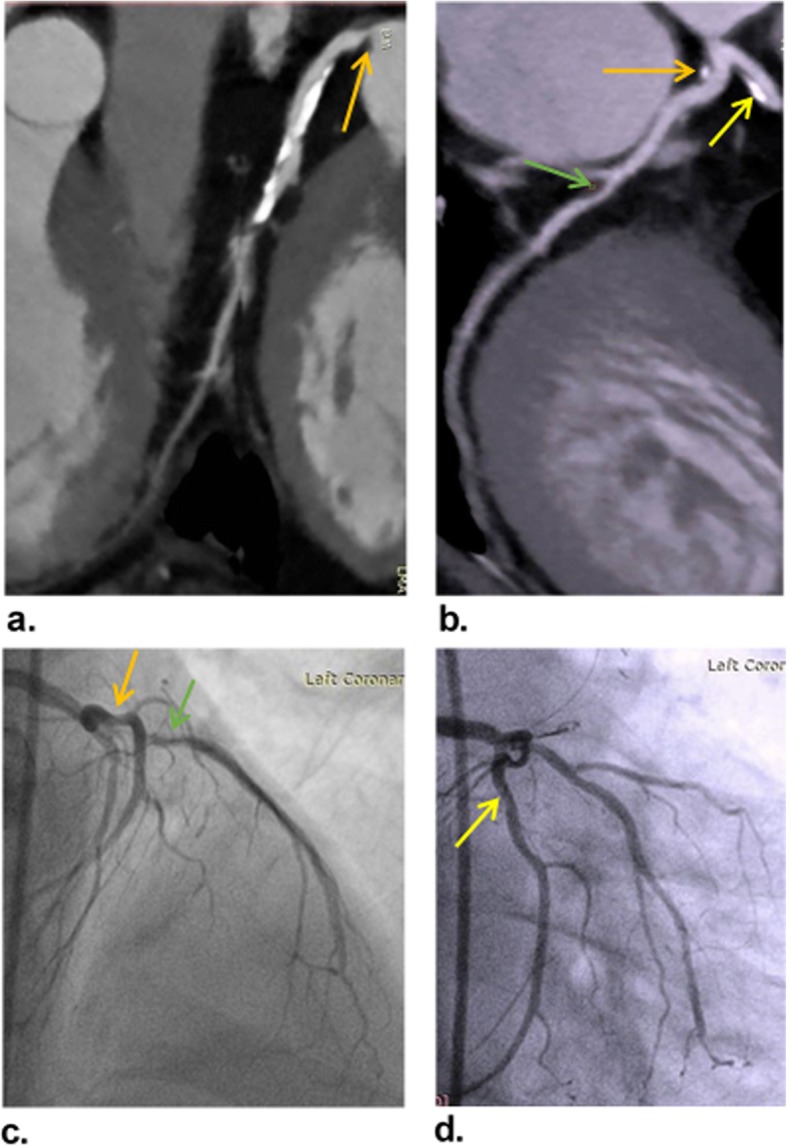
A 57-year-old man presented with typical chest pain. **a** and **b** Curved MPR images show mixed plaque in LMA with 57% diameter stenosis (orange arrows), another mixed plaque in LAD ostium with 25% diameter stenosis (yellow arrow), and soft plaque in mid LCX with 28% diameter stenosis (green arrow). The patient was categorized as CAD-RADS 4b. **c** and **d** ICA images confirm the non-significant stenosis of LAD (green arrow), LCX (yellow arrow), and LMA (orange arrow) with 25, 32, and 40% diameter stenosis, respectively, which are lower than the values detected on CCTA

**Fig. 5:**
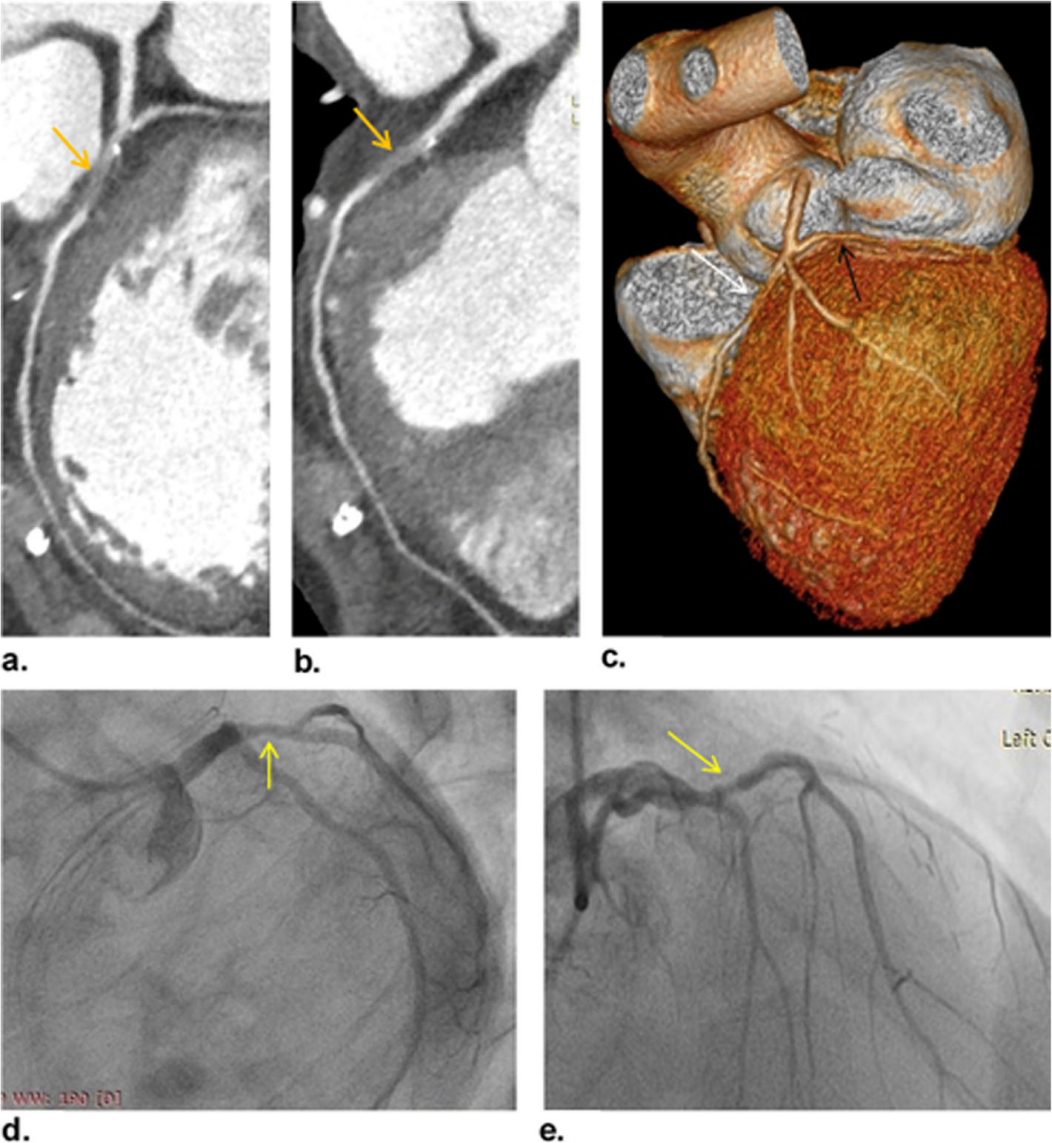
A 63-year-old man presented with typical chest pain. **a** and **b** Curved MPR images show total occlusion of LAD ostium (orange arrows), with distal retrograde filling of LAD and diagonal arteries by the collateral flow. **c** VR image shows mild proximal LCX stenosis (black arrow) and ostial LAD occlusion (white arrow) with distal refilling. The patient was categorized as CAD-RADS 5. **d** and **e** ICA images of LCA show patent LMA, proximal non-significant stenosis of LCX (yellow arrows), and non-opacification of LAD that is confirming total ostial occlusion diagnosed at CCTA

Representative cases of our study are shown in (Figs. 3, 4, and 5).

## Discussion

In an attempt to enhance the clinical impact and quality of medical imaging, structured reporting systems have become increasingly common, providing physicians with a direct relationship between objective information in the radiology report and evidence-based management recommendations [16]. The CAD-RADS classification is one of these reporting systems that have been emerged recently. A few numbers of recent studies have been published explaining the CAD-RADS in details and clarifying the pitfalls and limitations of this classification system [17–19]. However, since the pioneering report from Curye et al. in 2016, no study has been performed for external validation of the CAD-RADS. The current study is an attempt to validate the CAD-RADS and compare the results with ICA. We also assessed the application of such a reporting system for decision-making in clinical practice. The overall results are encouraging and show that this reporting system has excellent performance for categorization of CAD and predicting patients with significant CAD. However, more importantly in our opinion, we found that this classification system was highly appreciated by treating consultants as they could understand better the report of the CCTA examination and make more clear to them the findings observed as well as they felt more confident to take clinical decisions with their patients.

In terms of diagnostic validity, the CAD-RADS performed well, with a very high sensitivity, specificity, and accuracy (100%, 96.8 to 98.7%, and 98.3 to 99.3%, respectively, depending on the reviewer). This result is not surprising taking into consideration that it is based on CCTA findings, which have been extensively verified in many recent studies [20–22], and proven to be a robust modality for CAD diagnosis and determining the severity of coronary artery stenosis. However, the relatively high prevalence of significant CAD (45.6%) in our study might be a potential selection bias which could affect the calculation of sensitivity and specificity. Moreover, this high prevalence of significant CAD could explain the substantially higher positive predictive values in our study (96.3–98.5%) than those reported in most previous CT studies. The higher prevalence of significant CAD in our patient group may be explained by the collection of our patients from a large central institution which received complicated patients. The higher sensitivities and higher negative predictive values in our study were attributed to many factors: First, we used a dedicated Philips workstation with specific software able to eliminate the calcium. Second, we excluded patients with high calcium score. Third, we excluded patients with a bad image quality (patients with arrhythmia or irregular heart rate, and patients unable to sustain a breath-hold). Fourth, we excluded patients with non-diagnostic examination either due to motion/respiratory artifacts or calcific plaques hindered full plaque assessment. Fifth, we reviewed all images by highly experienced reviewers.

The CAD-RADS is easy to understand and apply. However, one of the most critical limitations of the present study was that CAD-RADS is still uncommon and unfamiliar by many clinicians. Consequently, the rationale and goal of the CAD-RADS have to be clarified to referring consultants in several scientific meetings before the start of the study. We found more encouraging results about how referring consultants evaluated this reporting system and its utility for clinical decision-making. Our simple survey among referring consultants involved in patient management showed that 87.5% of referring consultants considered CAD-RADS a helpful classification system for clinical decision-making and referral. Moreover, the three consultants who did not encourage the use of CAD-RADS in their management protocol attributed that to the novelty of this reporting system and time required for learning and training.

Without better evidence for reproducibility of CAD-RADS, the results of the study become unusable for clinical practice, and we remain uncertain whether this is a reporting system we should be applied or not. So, we performed inter-reviewer reproducibility in our study. The overall results were considered highly satisfactory. Our reporting of CAD-RADS categories and modifiers showed excellent IRA (ICC = 0.9862 and 0.8064, respectively). These results are very similar to that of the previous two studies [23, 24], which assessed the inter-observer agreement of CAD-RADS. However, as regards vulnerable plaque features (modifier “V”), the IRA was moderate (ICC = 0.5453). This finding is in line with the recent literature [25], which reports moderate reproducibility and high variability among readers regarding vulnerable plaque features. As the proper assessment of vulnerable plaque features on CCTA is still challenging, a further modification of CAD-RADS classification regarding the “V” modifier is mandatory.

Although ICA is the gold standard for the diagnosis of CAD, it is not risk-free, and the costs are considerable. Most of the related complications are mild, but even in the absence of severe CAD, critical complications may also occur. So, the non-invasive investigation may be the most convenient method for the detection of intermediate lesions [26]. Integrating CAD-RADS-guided recommendations into clinical practice may help to reduce much-referral for ICA and encourage further appropriate follow-up care for patients undergoing a diagnostic workup of CAD. This is very important in stable patients with intermediate lesions (CAD-RADS 3) as we identified high rates of early ICA in these patients soon after CCTA and likely before adequate trials of medical therapy.

Using the ROC curve, all the reviewers in this study strongly agreed that the optimal cutoff value of CAD-RADS for predicting patients with significant CAD was < CAD-RADS 3. This cutoff value was associated with 100% sensitivity. However, we were not capable of comparing our cutoff value as no available previous studies provided cutoff value for the CAD-RADS. Thus, we recommend further similar studies with a larger population to confirm or refute our cutoff value.

Finally, based on our findings, and in keeping with reports of previous authors, the CAD-RADS classification is considered a categorized reporting of CCTA findings with several advantages. Therefore, we strongly encourage the incorporation of CAD-RADS into CCTA reports. Nevertheless, the CAD-RADS still has drawbacks as some essential data are not listed in the assignment of CAD-RADS categories (e.g., myocardial CT perfusion and fractional flow reserve, non-coronary cardiac and extra-cardiac CT findings and interaction with other established reporting tools, Agatston calcium score, pretest probability, and coronary artery anomalies). Therefore, the CAD-RADS needs further modification to become accurate, useful, and comprehensive of all relevant descriptors and definitions. Future large longitudinal studies on the long-term clinical outcomes are still required to show the added value of these data to CAD-RADS classification and the concepts of referring clinicians on this reporting system.

There were some limitations to the study. First, this study was performed in a single center. Therefore, confirmation by larger multi-center studies is required. Second, the higher prevalence of significant CAD in our study might be a potential selection bias which could affect the calculation of sensitivity and specificity. Third, all CCTA examinations were analyzed by experienced reviewers; this is potentially affecting diagnostic performance. Thus, further studies about the performance of this reporting system when applied by non-experienced reviewers are needed. However, we observed that the reproducibility of this reporting system was high. Fourth, a CAD-RADS management protocol was provided to referring consultants before initiating the study. This could have biased their decision on how patients should be managed. Fifth, the exclusion of patients who were classified as CAD-RADS N from the study may represent a source of bias. Sixth, our study has a high number of CAD-RADS modifiers. The interpretation of CCTA in patients with previously known CAD needs high experience, especially in the intermediate lesions and in-stent restenosis that may be overestimated. However, all reviewers in our study had high experience. Finally, the CAD-RADS is still uncommon and unfamiliar by many clinicians and is still under modification.

## Conclusion

The CAD-RADS is valuable for improving CCTA structural reports with high diagnostic accuracy and high reproducibility, and very helpful for referring consultants for clinical decision-making.

## Data Availability

The datasets used and/or analyzed during the current study are available from the corresponding author on reasonable request.

## Abbreviations

AUC: Area under the curve
CAD-RADS: Coronary artery disease reporting and data system
CAD: Coronary artery disease
CCTA: Coronary computed tomography angiography
CI: Confidence interval
G: Graft
ICA: Invasive coronary angiography
ICC: Intra-class correlation
IRA: Inter-reviewer agreement
MIP: Maximum intensity projection
MPR: Multiplanar reformation
ROC: Receiver operating characteristic
S: Stent
SCCT: Society of cardiovascular computed tomography
V: Vulnerability
VRT: Volume rendering technique
